# Investigation of the human metabolism and disposition of the prolyl hydrolase inhibitor daprodustat using IV microtracer with Entero‐Test bile string

**DOI:** 10.1002/prp2.1145

**Published:** 2023-10-26

**Authors:** Guoying Tai, Fangming Xia, Cathy Chen, Adrian Pereira, Jill Pirhalla, Xiusheng Miao, Graeme Young, Claire Beaumont, Liangfu Chen

**Affiliations:** ^1^ Drug Metabolism and Pharmacokinetics GSK Collegeville Pennsylvania USA; ^2^ Drug Metabolism and Pharmacokinetics GSK, Stevenage Hertfordshire UK; ^3^ Drug Metabolism and Pharmacokinetics GSK, Ware Hertfordshire UK; ^4^ Present address: City of Hope Duarte California USA

**Keywords:** daprodustat, excretion, metabolism, metabolite structural identification, oral absorption, quantitative characterizations, radioactivity

## Abstract

Daprodustat is an oral small molecule hypoxia‐inducible factor (HIF) prolyl hydroxylase inhibitor (PHI) approved in Japan and the United States for the treatment of anemia associated with chronic kidney disease. This phase 1, nonrandomized, 2‐period, crossover study in 6 healthy men characterized and quantified the metabolites generated after a microtracer IV infusion of 50 μg (125 nCi) [^14^C]‐daprodustat administered concomitantly with a nonradiolabeled therapeutic dose of a 6‐mg daprodustat tablet, followed by a single oral solution dose of 25 mg (62.5 μCi) [^14^C]‐daprodustat. High‐performance liquid chromatography (HPLC) coupled with radioactivity detection (TopCount or AMS) and HPLC‐tandem mass spectrometry (HPLC‐MS^n^) were used for quantitative measurement and structural identification of radioactive metabolites in plasma, urine, feces, and bile. Following oral administration of [^14^C]‐daprodustat, unchanged daprodustat was the principal circulating drug‐related component, accounting for 40% of plasma radioactivity. Predominant oxidative metabolites M2, M3, M4, and M13 individually represented 6–8% of the plasma radioactivity and together accounted for the majority of radioactivity in urine and feces (53% in both matrices; 12% and 41% of dose, respectively). Unchanged daprodustat was not detected in urine and was only 0.7% of total radioactivity in feces (<0.5% of dose), with the remainder of the dose accounted for by oxidative metabolites. The radio‐metabolic profile of duodenal bile following IV infusion of [^14^C]‐daprodustat was similar to that observed in feces after oral administration. The data suggested that oral daprodustat was extensively absorbed, cleared exclusively by oxidative metabolism, and eliminated via hepatobiliary (primary) and urinary (secondary) excretion.

## Graphical abstract

Oral daprodustat undergoes oxidative metabolism exclusively and is eliminated via hepatobiliary and urinary excretion.

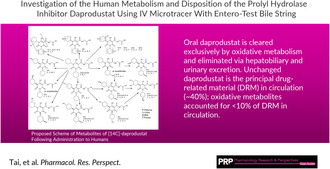

**Abbreviations**
ADME: absorption, distribution, metabolism, and excretion
AMS: accelerator mass spectrometry
AUC: area under the curve
CKD: chronic kidney disease
DPM: disintegrations per minute
DRM: drug‐related material
EPO: erythropoietin
HIF: hypoxia‐inducible factor
HPLC: high‐performance liquid chromatography
HPLC–MS^n^
: HPLC‐tandem mass spectrometry
LC‐MS^n^
: liquid chromatography–tandem mass spectrometry
LLQ: lower limit of quantification
LSC: liquid scintillation counting
PHI: prolyl hydroxylase inhibitor
TP1: treatment period 1
TP2: treatment period 2

## 1 INTRODUCTION

Anemia is common in patients with chronic kidney disease (CKD). Mechanisms contributing to anemia in these patients include deficiency of erythropoietin (EPO), a hormone that is produced in kidneys and to a lesser extent in the liver that supports normal red blood cell production, uremia‐induced inhibition of hematopoiesis, shortened erythrocyte survival, and disordered iron metabolism.^1^ The correction of anemia currently relies on use of intravenous erythropoiesis‐stimulating agents and iron administration, however, recombinant human EPO and its analogues have been associated with increased risk of adverse cardiovascular events.^2^, ^3^

Daprodustat is an orally available small molecule hypoxia‐inducible factor (HIF) prolyl hydroxylase inhibitor (PHI) approved in Japan and the United States in 2020 and 2023, respectively, for the treatment of anemia associated with CKD.^4^, ^5^, ^6^, ^7^ Daprodustat and other HIF‐PHIs stimulate erythropoiesis by inhibiting the HIF‐prolyl hydroxylase domain enzymes PHD1, PHD2, and PHD3.^8^, ^9^ Stabilization of HIF increases transcription of HIF‐responsive genes^10^ and stimulates components of the body's normal response to hypoxia, including increased EPO and hemoglobin production.^11^ In addition to induction of erythropoiesis by upregulation of EPO gene expression, HIF activation may also promote iron uptake and metabolism, and stimulates hematopoietic stem cells to differentiate into red blood cells.^12^, ^13^

Following single oral dosing of [^14^C]‐daprodustat to healthy male subjects, ~74% of radioactivity was recovered in feces, and ~ 20% in urine.^6^ In addition to the primary objectives of the clinical study^6^ the purpose of the current investigations was to comprehensively conduct quantitation and structural characterization of daprodustat and its metabolites in plasma, urine, and feces following single oral administration of daprodustat to humans. An additional objective was to characterize daprodustat and its metabolites in duodenal bile by sampling with Entero‐Test bile strings, following intravenous infusion of radiolabeled daprodustat dosed concomitantly with an oral tablet at a therapeutic dose. Lastly, attempts were made to compare and integrate the results of circulating metabolites from the current single‐dose radiolabel study in healthy subjects with those reported previously following repeat‐dose administration of nonradiolabeled daprodustat in patients with CKD.^14^ Overall, the intent was to establish a more complete profile of daprodustat absorption, metabolism and excretion, and gain better understanding of clearance mechanisms in daprodustat disposition and elimination.

## 2 METHODS AND MATERIALS

### 2.1 Subjects and study design

The design and clinical pharmacokinetics of daprodustat in the crossover mass balance study in 6 healthy male participants have been previously described.^6^ Briefly, the study consisted of 2 treatment periods 2 weeks apart. During treatment period 1 (TP1) each participant was administered 50 μg/125 nCi (4.6 MBq) of [^14^C]‐daprodustat in sterile isotonic phosphate buffer intravenously concomitantly with an oral nonradiolabeled 6‐mg daprodustat tablet. During treatment period 2 (TP2), 25 mg/62.5 μCi (2.3 MBq) of [^14^C]‐daprodustat in sterile phosphate buffer was administered orally. Here, we report additional data on the metabolism and disposition of daprodustat following both routes of administration, with the addition of duodenal bile collection for metabolite characterization. The clinical study was approved by an independent ethics committee and was conducted according to the recommendations of Good Clinical Practice and the Declaration of Helsinki.^6^ All subjects provided written informed consent to participate in the study.

### 2.2 Chemicals and reagents

[^14^C]‐Daprodustat/GSK1278863G (radiochemical purity of >98.7% and specific activity of 2.51 μCi/mg, equivalent to 0.093 MBq/mg) was synthesized by Isotope Chemistry (GSK, Stevenage, UK). A single [^14^C] label was positioned in the pyrimidine moiety. Unlabeled daprodustat and metabolite standards, metabolite M2/GSK2391220 (Batch Number: H11454‐120‐1A, purity 95%), M3/GSK2506104 (Batch Number: ES2553‐60, purity 99%), M4/GSK2487818 (Batch Number: H10694‐064, purity 92%), M5/GSK2506102A (Batch Number: H10694‐046, purity 94%), M6/GSK2531398 (Batch Number: H10694‐058, purity 92%), and M13/GSK2531401 (Batch Number: H11451‐114‐A1, purity 97%) were supplied by Jurong GSK (Singapore) or Wuxi AppTech (Shanghai, China). Chemicals and solvents of reagent or high‐performance liquid chromatography (HPLC) grade were purchased from commercial sources.

**FIGURE 1:**
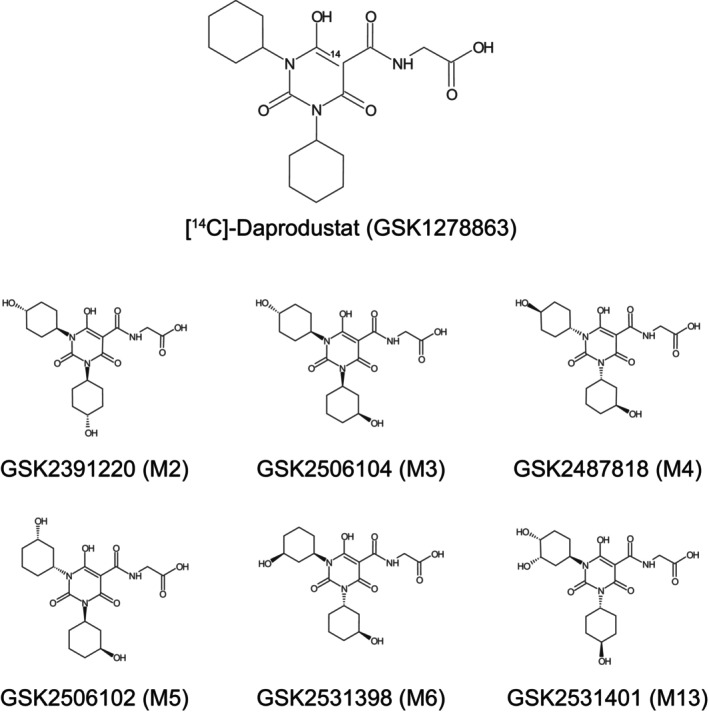
Chemical Structures of Daprodustat and Metabolites. Structures represent the predominant single stereoisomeric form of human circulating metabolites M2, M3, M4, M5, M6, and M13.^29^

Structures of the reference standards are shown in Figure 1.

### 2.3 Collection of samples

Duodenal bile samples were collected for metabolite characterization on Entero‐Test bile strings, a commercially available noninvasive procedure to collect upper gastrointestinal fluid.^15^ In TP1, samples were collected at 1 h post‐^14^C‐IV dose (3 h postoral tablet dose) for qualitative assessment of biliary metabolites. In TP2, samples for metabolite quantification and characterization included blood samples that were collected predose, and at 0.5, 1, 1.5, 2, 3, 4, 6, 8, 10, 12, 24, 36, 48, 72, 96, 120, and 144 h postoral dose, and urine and feces that were collected predose and at 24 h intervals up to 168 h postdose if available.

### 2.4 Sample pooling and pretreatment

#### 2.4.1 Plasma

Two pooled plasma samples from TP2 were prepared from the six human subjects based on total radioactivity in the samples. A primary pool comprising approximately 95% of the total plasma radioactivity was created according to the area under the response‐time curve (AUC) concentration‐time proportional pooling method.^16^ Considering that the terminal phase half‐life (t_½_) is approximately 2 h for daprodustat and the six predominant metabolites,^6^ for each subject, aliquots of plasma samples collected at each time point between 0 and 8 h postdose were pooled in volumes proportional to time intervals between sampling time points. Equal volumes of the individual pools from each subject were then pooled to create one plasma sample that was representative of the mean AUC over the range of 0 to 8 h. The second pool was generated by mixing equal volumes of the plasma samples at 10 and 12 h across all subjects. Radioactivity in the plasma pools was determined by liquid scintillation counting (LSC).

The two plasma pools were subjected to protein precipitation cleanup in three serial steps of methanol/acetonitrile extraction and centrifugation. Resultant supernatants were combined and evaporated to near dryness under a stream of nitrogen. Remaining residues were reconstituted in methanol/water (1:4) at a concentration factor of 5. Radioactivity recovery and concentration following sample cleanup was determined by LSC prior to subsequent quantitative radio‐HPLC analysis.

#### 2.4.2 Urine

Urine samples from TP2 were analyzed without pooling since >95% of cumulative urinary dose excretion occurred during the first 24 h.^6^ Aliquots of urine samples collected in the first time interval (0–24 h) from each human subject were centrifuged and subsequent recovery of radioactivity was determined by LSC prior to quantitative radio‐HPLC analysis.

#### 2.4.3 Feces

Weighed aliquots of selected fecal homogenates from TP2 were pooled in proportion to total sample weight for each human subject to include the majority of excreted radioactivity. The time range of the pooled fecal homogenates varied from 24 to 120 h postdose, among subjects, depending on the rate of radioactivity excretion. Radioactivity of the pooled fecal homogenates was determined by LSC. Each pooled fecal homogenate was extracted twice with methanol/ammonium formate pH 3.0 and the resulting two supernatants were combined, evaporated, and reconstituted in methanol/water (1:3). Sample preparation recovery of radioactivity was determined by LSC prior to subsequent quantitative radio‐HPLC analysis.

#### 2.4.4 Duodenal bile

To assess the drug‐related material (DRM) arising from the radiolabeled intravenous dose (TP1), individual bile strings were extracted with either acetonitrile or water in two separate extraction procedures. An aliquot of 500 μL from each acetonitrile or water extract was pipetted into a scintillation vial and mixed with 15 mL ScintiLogic U cocktail. An equal volume of the opposite solvent to which the extraction was conducted (i.e., either water or acetonitrile) was also added.

All scintillation vials were stored in the dark at approximately 4°C for at least 24 h, briefly mixed, and counted for 60 minutes on a low‐level LSC to determine radioactivity content.

Based on the levels of extracted radioactivity, equal volume aliquots of 100 μL from three subjects were pooled and spiked with 1200 μL of a cold standard mix solution in water to create a single diluted pool of bile string extracts at approximately 15 DPM/mL for HPLC followed by analysis by accelerator mass spectrometry (AMS). Preparation of the cold standard mix solution is described in Data S1 (Supplemental Item 1).

A concurrent inline UV‐chromatogram was obtained before fractions were collected for analysis by AMS so that the retention times for the radioactive peaks for daprodustat and the six metabolites (M2–6 and M13) for which authentic standards were available could be verified.

To assess DRM arising from the nonlabeled oral dose, the remaining bile extracts (pooled and individual) were prepared into a single sample as described below, for metabolite identification by liquid chromatography–tandem mass spectrometry (LC–MS^n^). All remaining bile string extracts were further pooled to create a single pool, dried under a stream of nitrogen, and reconstituted in 150 μL of methanol/water (1:4) to achieve 40 times concentration factor. The concentrated bile extract was centrifuged at approximately 14 000 g for 10 min and the supernatant was aliquoted for analysis by LC‐MS^n^ analysis. Fractions were collected during analysis by LC‐MS^n^ for subsequent generation of a radio profile to assist with peak assignment in the bile extract in a comparable way to that described in the LC‐MS^n^ method investigations.

### 2.5 Quantitative radio‐HPLC analysis

Radio‐HPLC analysis was conducted on selected urine, fecal, and plasma samples on a 1200 HPLC system (Agilent Technologies, Palo Alto, CA). Radio‐HPLC conditions are described in Data S1 (Supplemental Item 2).

Urine and feces samples were injected onto the HPLC and the column eluate from each sample was fractionated into a set of four LumaPlate‐96 microplates at 12 s/well. The mobile phase solvent in microplate wells was evaporated to dryness. The dried plates were sealed and counted on a TopCount scintillation counter for 5 or 10 min/well. The counting data were processed in Laura v4.1 to generate radiochromatograms.

Plasma samples were analyzed in a similar manner to that of the urine and feces samples except that significant steps were taken to maximize radioactive signal intensity as follows: (1) each plasma extract was injected up to 8 times, (2) the resulting column eluate from each injection was cumulatively collected into the same set of microplates, and (3) after the last injection, the dried microplates were counted for 30 min/well.

Each peak on a radiochromatogram was calculated as a percentage of the total count detected and expressed as nanogram equivalents of daprodustat per gram of plasma or the percentage of the dose recovered in urine and feces (all corrected for the overall recovery of extraction and reconstitution). A Topcount peak height of 10× standard deviation of background counts was defined as the lower limit of quantification (LLQ).

[^14^C]‐daprodustat‐spiked stability control samples of plasma, urine, and feces were prepared and analyzed as described above to monitor any potential degradation of the parent compound during sample storage and handling.

To deconvolute and estimate concentrations of M2 and M33, which coeluted in the major radio peak, the concentration values of M3, M4, and M6 determined using a validated LC/MS bioanalytical method^6^ were first virtually pooled in the same manner as in the 0–8 h plasma pool to derive a pooled concentration for each. The ratios (and the mean of the ratios) of the radiolabeled quantification data over the derived bioanalysis concentration data were then calculated for M3, M4, and M6. Lastly, the same virtual pooling approach was conducted for M2 and the above mean ratio was applied to estimate the levels of M2 and M33, respectively.

### 2.6 HPLC‐UV+AMS analysis for bile samples

The pooled bile string extract solution was spiked with nonradiolabeled standards, mixed and injected (100 μL), then chromatographed by HPLC. The HPLC column eluate was fractionated (12‐s intervals between 15 and 55 min) and collected into prebaked small quartz tubes containing copper oxide wire.

Additionally, replicate aliquots of the injection solution of the spiked pool (4 × 10 μL) were separately pipetted into the same type of quartz tubes as stated above for the determination of HPLC column recovery of injected radioactivity.

Analysis by AMS requires conversion of samples via a two‐step process of oxidation to carbon dioxide (CO_2_) and then reduction to graphite.^17^ AMS provides an isotope ratio [^14^C]/[^12^C] from which ^14^C per mg carbon is derived.^18^ Additional details of HPLC‐UV+AMS analysis for bile samples are described in Data S1 (Supplemental Item 3).

### 2.7 Mass spectrometric analysis

LC‐MS^n^ was used to analyze representative samples of plasma and bile extracts and urine and fecal homogenate extracts according to the LC conditions described previously. During the LC separation, a postcolumn split was used to direct approximately 15% of the flow to an LTQ‐Orbitrap XL (ThermoFisher, San Jose, CA) mass spectrometer equipped with an electrospray ionization source in positive‐ion mode, implementing data‐dependent scanning by using a mass list containing masses of all known and probable metabolites. A full‐scan mass spectrum (at resolution 30 000 for the Orbitrap) was collected and the data interrogated in real time to identify mass peaks corresponding to masses in the mass list. If present, the mass peaks were selected as target peaks for subsequent MS^n^ scans. The remaining LC eluate from the postcolumn split was directed into scintillator‐coated microtiter plates (using a LEAP HTS PAL fraction collector, Leap Technology, Carrboro, NC) or MicroBeta plates, or sent to waste. Fractions were dried and the radioactivity in each well was counted on a PerkinElmer Topcount scintillation counter or a PerkinElmer MicroBeta2 model 2450 counter. The counting data were processed in Laura v4.1 to generate radiochromatograms followed by manual integration of radio peaks. Data were acquired and processed using Xcalibur software (version 2.1; Thermo Scientific, Wal). Predose matrix samples of plasma, urine, and feces were prepared as described above, and analyzed by LC‐MS^n^ to aid in distinguishing between DRMs and endogenous components.

### 2.8 Nomenclature of targets and ligands

Key protein targets and ligands in this article are hyperlinked to corresponding entries in http://www.guidetopharmacology.org, the common portal for data from the IUPHAR/BPS Guide to PHARMACOLOGY,^19^ and are permanently archived in the Concise Guide to PHARMACOLOGY 2019/20.^20^

## 3 RESULTS

### 3.1 HPLC column recovery

HPLC column recoveries of selected urine (TP2), selected feces (TP2), and duodenal bile extracts (TP1) were essentially complete, indicating no or minimum loss of radioactivity during chromatographic separation (Table 1).

**TABLE 1:** The retention times and fragment of all metabolites in human plasma, urine and bile samples.

Metabolite	*t*_R_ (min)	[M+H]^+^	*m*/*z*
			Typical MS^n^ ion fragments
Daprodustat	51.6	392	MS^2^: 291 MS^3^: 122, 166, 209, 223
M2	18.2	424	MS^2^: 323 MS^3^: 138, 157, 182, 225, 255
M3	20.3	424	MS^2^: 323 MS^3^: 138, 140, 182, 225, 255
M4	21.1	424	MS^2^: 323 MS^3^: 138, 140, 157, 182, 225, 255
M5	21.9	424	MS^2^: 323 MS^3^: 138, 140, 182, 225, 255
M6	22.8	424	MS^2^: 323 MS^3^: 138, 140, 182, 225, 255
M7	24.2	424	MS^2^: 323 MS^3^: 138, 157, 182, 225, 255
M8	33.3	408	MS^2^: 307 MS^3^: 122, 138, 166, 182, 209, 225, 239
M9	34.4	408	MS^2^: 307 MS^3^: 122, 138, 140, 182, 209, 225, 239
M10	37.1	408	MS^2^: 307 MS^3^: 122, 138, 140, 182, 209, 225, 239
M13	15.7	440	MS^2^: 339 MS^3^: 138, 182, 225, 241, 271, 321
M14	22.0	422	MS^2^: 225, 321 MS^3^: 225
M15	23.7	422	MS^2^: 225, 321 MS^3^: 225
M16	25.2	422	MS^2^: 225, 321 MS^3^: 225
M22	34.4	408	MS^2^: 307 MS^3^: 122, 140, 166, 209, 225, 239
M30	14.0	440	MS^2^: 339 MS^3^: 138, 182, 225, 241, 271, 321
M31	15.4	440	MS^2^: 339 MS^3^: 138, 182, 225, 241, 271
M32	18.0	440	MS^2^: 339 MS^3^: 138, 182, 225, 241, 271, 321
M33	18.8	440	MS^2^: 339 MS^3^: 138, 182, 225, 241, 271, 321

Abbreviations: [M+H]^+^, protonated molecule; *m*/*z*, mass‐to‐charge ratio; MS^n^, tandem mass spectrometrya; *t*_R_, retention time.

[^14^C]‐daprodustat‐spiked stability control samples of plasma, urine, and feces showed no sign of degradation during sample storage and treatments.

### 3.2 Radio‐HPLC profiles

#### 3.2.1 Plasma (TP2)

The levels of total radioactivity in the 0–8 h and 10–12 h plasma pools were 1492 DPM/g and 121 DPM/g, respectively, corresponding to 268 ng daprodustat equivalents/g and 22 ng daprodustat equivalents/g. Overall recovery of radioactivity following sample preparation was 85 and 69% for the 0–8 h and 10–12 h pools, respectively. The low recovery in the 10–12 h sample was attributed to a very low initial level of radioactivity, as quantified by LSC (rather than by AMS).

**FIGURE 2:**
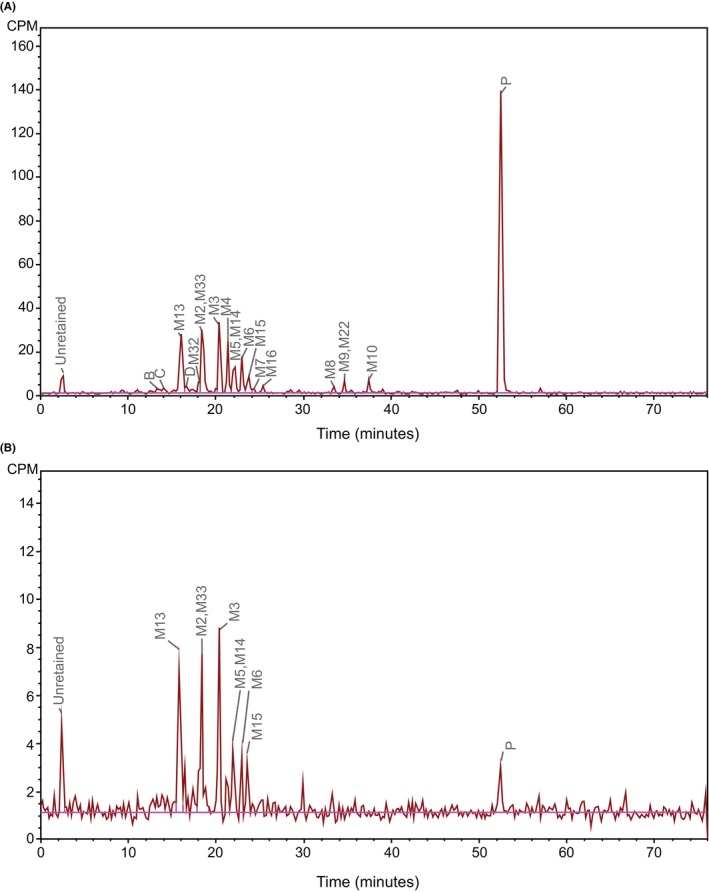
Reconstructed HPLC‐radiochromatograms of pooled plasma extracts following a single oral dose of [^14^C]‐daprodustat at 25 mg/62.5 μCi. (A) 0–8 h pool (B) 10–12 h pool. CPM, counts per minute; P, parent drug (daprodustat). B, C, and D are unassigned radio peaks. Multiple HPLC injection and cumulative microplate collection for metabolite profiling of [^14^C]‐daprodustat in human plasma. To allow for radio‐profiling analysis of more than a dozen circulating metabolites of daprodustat on a conventional TopCount microplate counter, up to eight aliquots of a plasma extract were injected on an HPLC and the resulting column eluates were cumulatively collected onto a set of four 96‐well scintillate‐coated microplates. Briefly, following an HPLC injection and column eluate collection, the microplates were partially evaporated to accommodate the subsequent fraction collection. The cycle from a sample injection to partial evaporation was repeated until the last HPLC run where the plates were brought to a complete dryness for [^14^C] counting. To ensure paramount reproducibility of the multiple HPLC runs, the HPLC system was kept running continuously. A solution of non‐radiolabeled standards of daprodustat and its major metabolites, used as on‐the‐go UV retention time markers, was analyzed presequence of plasma injections and after each injection of the plasma sample.

Quantitative results of radioactive components in pooled plasma samples are given in Table 2, expressed as percentage of plasma radioactivity and nanogram equivalents of daprodustat per gram of plasma. Reconstructed HPLC‐radiochromatograms of 0–8 h and 10–12 h pooled plasma extracts are shown in Figure 2. In the 0–8 h plasma pool, the principal circulating drug‐related component was unchanged daprodustat, representing 40% of the plasma radioactivity, or 106 ng/g (ng equivalents daprodustat/g plasma). Sixteen metabolites were identified in plasma, most of which were formed by mono‐, di‐, or tri‐oxidation of the cyclohexane ring(s), and these together accounted for approximately 51% of plasma radioactivity. A major radio peak (coeluting M2 and M33) accounted for 10% of plasma radioactivity. Other predominant metabolites, M3, M4, and M13, accounted for 8%, 6%, and 8% of plasma radioactivity, respectively. These percentages were consistent with the levels determined using the validated multianalyte (parent and six metabolite) bioanalytical assay.^6^ The respective levels of M2 and M33 were deconvoluted using the mean ratio of M2 to M3, M4, and M6 derived from the validated bioanalytical assay, as described in Methods and Materials. M2 was estimated to be 8% of plasma radioactivity with M33 being approximately 2% in the 0–8 h pool. This deconvolution exercise was not conducted for the 10–12 h pool due to its very low total radioactivity. All other metabolites (M5, M6, M9, M10, M14, M15, M22, and M32) and an unassigned radio peak D were detected, individually at <5% of plasma radioactivity.

**TABLE 2:** Quantification of the radioactive components in plasma, urine, and fecal extracts following a single oral dose of [^14^C]‐daprodustat and relative percentage of radioactive components in duodenal bile string extract following an intravenous infusion of [^14^C]‐daprodustat (concomitant to an oral dose of daprodustat).

Peak	% Radioactivity in plasma^a^ (ng equivalents daprodustat/g plasma^b^)		Mean % radioactivity in urine and feces^a^ (mean % administered dose^c^)		% Radioactivity in bile^d^
	0–8 h	10–12 h	Urine	Feces	
Daprodustat	39.5 (106)	<LLQ (<LLQ)	ND (ND)	0.7 (0.5)	0.5
M2 and M33 (only M2 in feces)	10.4^e^ (27.7)	14.4 (3.1)	15.8 (3.2)	19.8 (14.4)	12.3
M3	7.6 (20.2)	11.8 (2.6)	16.0 (3.3)	14.1 (10.3)	20.0
M4	5.7 (15.2)	ND (ND)	7.9 (1.6)	17.0 (12.3)	15.5
M5 and M14	4.5 (11.9)	<LLQ (<LLQ)	10.4 (2.1)	7.7 (5.6)	11.1
M6	3.6 (9.8)	<LLQ (<LLQ)	5.8 (1.2)	6.2 (4.5)	6.3
M7	<LLQ (<LLQ)	ND (ND)	0.5 (0.1)	2.2 (1.6)	3.1
M8	0.8 (2.2)	ND (ND)	0.2 (0.04)	3.3 (2.4)	3.9
M9 and M22	1.3 (3.5)	ND (ND)	1.5 (0.3)	1.9 (1.4)	2.4
M10	1.5 (4.0)	ND (ND)	<LLQ (<LLQ)	3.4 (2.5)	3.7
M13	8.3 (22.1)	16.1 (3.5)	16.5 (3.4)	4.9 (3.6)	2.4
M15	2.5 (6.8)	<LLQ (<LLQ)	4.8 (1.0)	1.9 (1.4)	ND (ND)
M16	0.9 (2.5)	ND (ND)	0.9 (0.2)	1.1 (0.8)	ND (ND)
M30	ND (ND)	ND (ND)	0.4 (0.1)	ND (ND)	ND (ND)
M31	ND (ND)	ND (ND)	0.4 (0.1)	1.3 (0.9)	1.2
M32	1.0 (2.6)	ND (ND)	3.2 (0.7)	0.5 (0.4)	ND (ND)
A	ND (ND)	ND (ND)	ND (ND)	1.3 (0.9)	ND (ND)
B	1.0 (2.5)	ND (ND)	2.2 (0.4)	2.5 (1.8)	ND (ND)
C	0.8 (2.2)	ND (ND)	ND (ND)	0.8 (0.6)	ND (ND)
D	1.2 (3.2)	ND (ND)	0.2 (0.04)	1.4 (1.1)	ND (ND)
E	ND (ND)	ND (ND)	0.2 (0.1)	ND (ND)	ND (ND)
F	ND (ND)	ND (ND)	ND (ND)	ND (ND)	2.3
G	ND (ND)	ND (ND)	ND (ND)	ND (ND)	2.4
H	ND (ND)	ND (ND)	ND (ND)	0.6 (0.4)	ND (ND)
Unretained radio peak	2.4 (6.4)	7.8 (1.7)	ND (ND)	ND (ND)	ND (ND)
Total quantified	92.8 (249)	50.0^f^ (10.9)	86.7 (17.7)	92.5 (67.4)	87.1
(% Dose in sample analyzed)	N/A	N/A	(20.5)	(72.8)	N/A
(% Dose in total sample)	N/A	N/A	(21.1)	(73.7)	N/A
LLQ (% matrix radioactivity)	1	5	1–2	<0.5	N/A

Abbreviations: DPM, disintegrations per minute; LLQ, lower limit of quantification; ND, not detected.

^a^ % radioactivity recovered under each integrated radio peak.

^b^ ng equivalents of daprodustat per g of plasma under each integrated radio peak.

^c^ Mean % administered dose recovered under each integrated radio peak.

^d^ Absolute quantification of drug‐related components is not possible due to the qualitative nature of the bile string sampling and extraction procedure.

^e^ The estimation for M2 and M33 was 8.1% and 2.3% respectively, based on extrapolation from validated bioanalytical data for M2, M3, M4, and M5.

^f^ Low value due to limited radioactivity in the sample for HPLC analysis (ca. 168 DPM on column).

In the 10–12 h pool, only metabolite peaks of coeluted M2 and M33, M3, and M13 were quantifiable, each representing 14%, 12%, and 16% of plasma radioactivity, respectively. Peaks M5, M6, M14, M15, and P were detected at below the LLQ.

Overall, quantifiable peaks constituted 93% and 50% of plasma radioactivity in the 0–8 h and 10–12 h pools, respectively.

#### 3.2.2 Urine (TP2)

**FIGURE 3:**
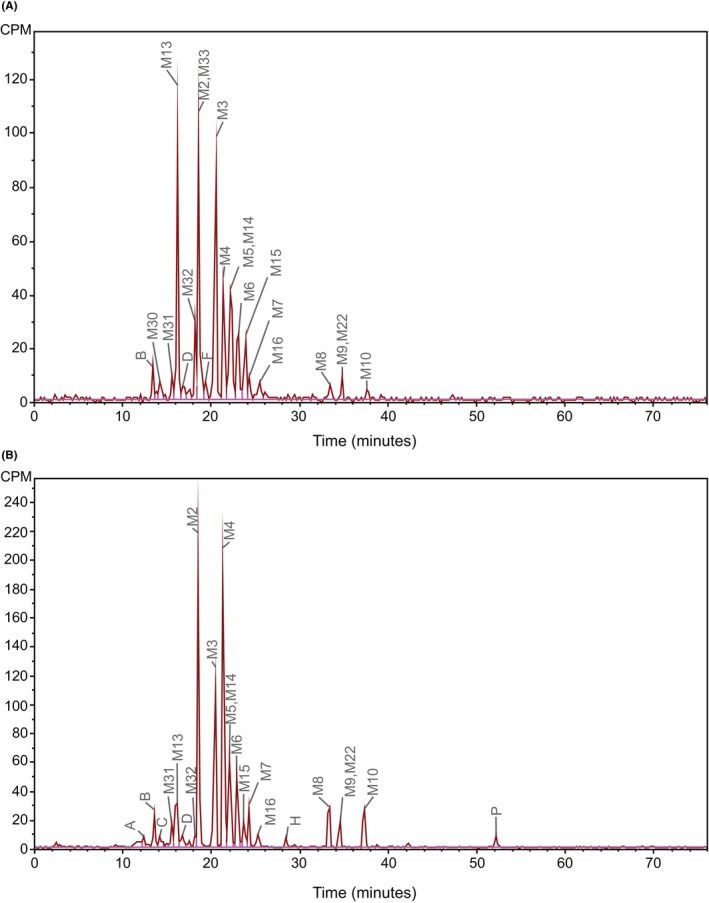
Representative reconstructed HPLC‐radiochromatograms of urine and fecal extracts from a single subject following a single oral dose of [^14^C]‐daprodustat at 25 mg/62.5 μCi. (A) Urine 0–24 h (B) Feces 48–96 h. CPM, counts per minute; P, parent drug (daprodustat). A, B, C, D, and H are unassigned radio peaks.

Recovery of urine radioactivity following centrifugation was complete, indicating no loss of drug‐related components during sample processing. The 0–24 h urine samples that were analyzed represented 97% of total cumulative radioactivity excreted in the urine. Minor qualitative differences in the urine radio profiles were observed across the 6 subjects. Figure 3A shows a representative reconstructed HPLC‐radiochromatogram of urine from a single subject.

Unchanged daprodustat was not detected in the 0–24 h urine from any subject. The metabolite profile in urine was similar to that in plasma. Predominant radio peaks in urine were M3, M13, a coeluting peak of M2 and M33, and a coeluting peak of M5 and M14, accounting respectively for a mean of 16%, 17%, 16%, and 10% of urine radioactivity, or 3%, 3%, 3%, and 2% of the administered dose. Additional metabolites M4 and M6 each accounted for a mean of 8% and 6% of urine radioactivity, or 2% and 1% of the dose, respectively. All other metabolites (M9, M15, M22, M32), an unassigned radio peak B, and several minor metabolites and unassigned radio peaks were detected, individually at <5% of urine radioactivity (≤1% of dose). Mean radioactivity quantified in urine was approximately 87% of the total present.

#### 3.2.3 Feces (TP2)

The analysis of pooled fecal homogenates showed that 99% of the total cumulative radioactivity in the excreta was recovered, indicating little loss of drug‐related components during sample preparation. As with urine, minor qualitative differences in the radio profiles of fecal extracts were observed across subjects. Figure 3B shows a representative reconstructed HPLC‐radiochromatogram from a single subject.

Unchanged daprodustat accounted for a mean of approximately only 0.7% of fecal radioactivity, or 0.5% of the administered dose (Table 2). Predominant metabolites in feces were M2, M3, and M4, accounting respectively for a mean of 20%, 14%, and 17% of radioactivity in feces, or 14%, 10%, and 12% of the administered dose. Additional radio peaks M6 and a coeluting peak of M5 and M14 accounted respectively for a mean of 6% and 8% of radioactivity in feces, or 5% and 6% of the dose. All other metabolites (M7, M8, M9, M10, M13, M15, M16, M22, M31, and M32) and unassigned radio peaks A, B, C, D, and H were detected individually at <5% of feces radioactivity (<4% of dose). The mean radioactivity quantified in feces was approximately 93% of the total present.

#### 3.2.4 Duodenal bile (TP1)

**FIGURE 4:**
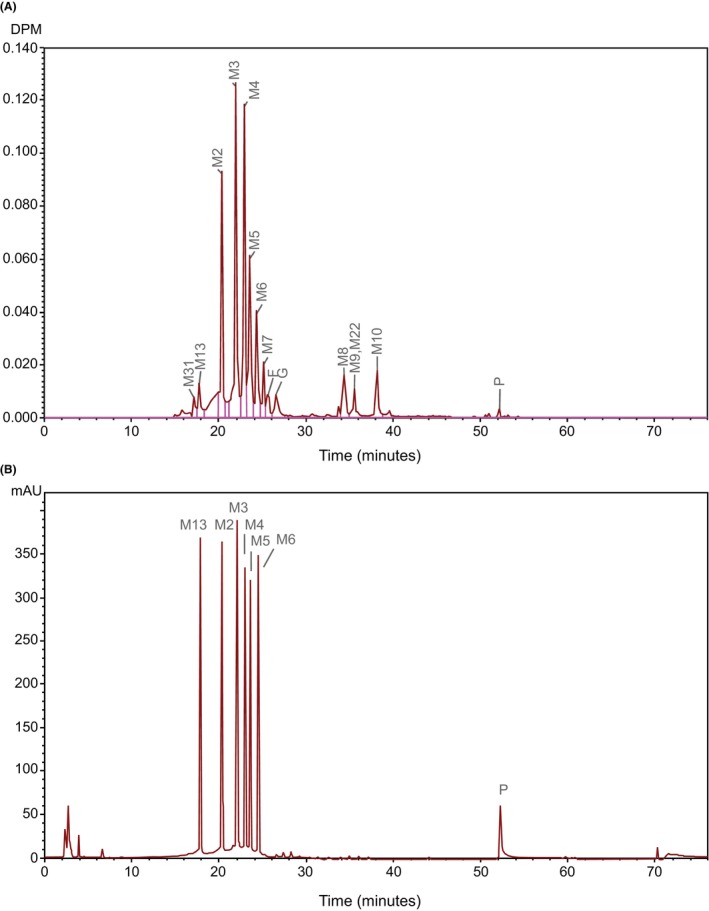
Reconstructed HPLC‐AMS radiochromatogram and UV‐chromatogram of pooled and diluted duodenal bile extract following a single intravenous Infusion of [^14^C]‐daprodustat at 50 μg/125 nCi. (A) Radiochromatogram. Data collection time range was 15–55 min. Radio peaks of M2, M13, and P were assigned by cochromatography. Other peaks were assigned by LC‐MS (B) UV‐chromatogram—showing retention time positions of co‐spiked authentic standards (daprodustat, M2‐6 and M13). DPM, disintegrations per minute; mAU, milli‐absorbance unit; P, parent drug (daprodustat). F and G are unassigned radio peaks.

Figure 4A shows a reconstructed HPLC‐AMS radiochromatogram of pooled bile string extract that had been diluted with standards and Figure 4B shows a UV‐chromatogram obtained inline from the same HPLC run to identify radio peaks using spiked metabolite standards. Relative percentages of radioactive metabolites in bile following intravenous infusion are given in Table 2.

Unchanged daprodustat accounted for only 0.5% of biliary radioactivity, indicating extensive hepatic metabolism of the parent compound. The metabolic profile in bile was comparable to that for urine and feces. The predominant metabolites in duodenal bile were M2, M3, M4, and M5, each accounting for 11% to 20% of bile radioactivity. The additional metabolite M6 peak accounted for 6% of bile radioactivity. A number of minor radioactive peaks M7, M8, M9, M10, M13, M22, and M31, as well as unassigned peaks F and G, each accounted individually for <4% of bile radioactivity. The quantified radioactivity in bile was approximately 87% of the total in the string extract pool.

## 4 DISCUSSION

Following a single oral dose of [^14^C]‐daprodustat (TP2) in healthy human subjects, unchanged daprodustat was the principal circulating DRM in 0 to 8 h plasma. Predominant circulating human metabolites M2, M3, M4, and M13 represented 6% to 8% of plasma radioactivity. Minor metabolites including M5, M6, M14, M15, and M33 were observed at levels individually representing <5% of plasma radioactivity. As stated in the Methods, two pooled samples from TP2 were prepared based on total radioactivity in the samples to provided robust quantitative data on the principle circulating drug‐related component and metabolites, a primary pool comprising approximately 95% of the total plasma radioactivity created according to the area under the response‐time curve (AUC) concentration‐time proportional pooling method^16^ and a second pool generated by mixing equal volumes of the plasma samples at 10 and 12 h across all subjects.

Consistently, previous steady‐state clinical data in patients with anemia associated with various stages of CKD also indicated that daprodustat was the principal circulating DRM in human plasma, measured at up to 55% by AUC ratio.^14^ M2 and M3 exposure were reported to be around 11% DRM based on AUC ratio while the level of other metabolites (M4, M5, M6, and M13) ranged from approximately 3% to 9% in subjects with normal kidney function previously.^14^ The slight differences noted in %DRM for M2 and M3 when compared with the human ADME study are likely because Caltabiano et al. calculated %DRM based on AUC ratio of each metabolite to the total AUC of parent drug plus only six predominant metabolites, whereas in the current investigation, all detectable metabolites were taken into account and %DRM was determined based on the radioactivity measurements. In fact, M2 and M3 would have both reached approximately 10% DRM in the current study if total radioactivity were only summed by parent and the six predominant metabolites after discounting all other detected radioactivity (metabolites) in plasma. Therefore, the human ADME study using radiolabeled drug provided comprehensive metabolite profiles in human plasma and enabled better quantitative metabolite assessment of all metabolites in healthy subjects, especially when availability of authentic standards is not possible for every circulating metabolite. It is worth noting that M2, M3, M13, and other predominant metabolites apparently had longer terminal half‐lives due to a potential effect of the severity of kidney disease on drug elimination, which subsequently resulted in exposure increases of two‐ to six‐fold in patients with CKD stage 3/4 or anemic subjects on either hemodialysis or peritoneal dialysis.^14^ Consequently, metabolites M2, M3, and M13 have been classified as major circulating human metabolites given that they represent >10% of plasma observed DRM in a clinically relevant setting (i.e., reaching steady‐state exposure in the target patient population of anemia associated with CKD). The three other circulating metabolites of daprodustat (M4, M5, and M6) were below 10% of DRM.^14^

Comprehensive safety evaluations of the three major metabolites have therefore been conducted, and adequate toxicology and safety qualifications established throughout the development program.^10^, ^21^ In this regard, rabbit and monkey were considered relevant nonclinical species for toxicology evaluation following administration of daprodustat as they produce metabolites with systemic exposure profiles similar to humans. Toxicology evaluations were also performed^21^ following direct subcutaneous administration of the three major circulating human metabolites (M2, M3, and M13) to mice and rats because these species produce no or limited metabolites of daprodustat. The disposition of daprodustat together with appropriate experimental approaches supports the use of mouse, rat, rabbit, and monkey as relevant nonclinical species for the evaluation of the safety of daprodustat for the proposed clinical uses.

**FIGURE 5:**
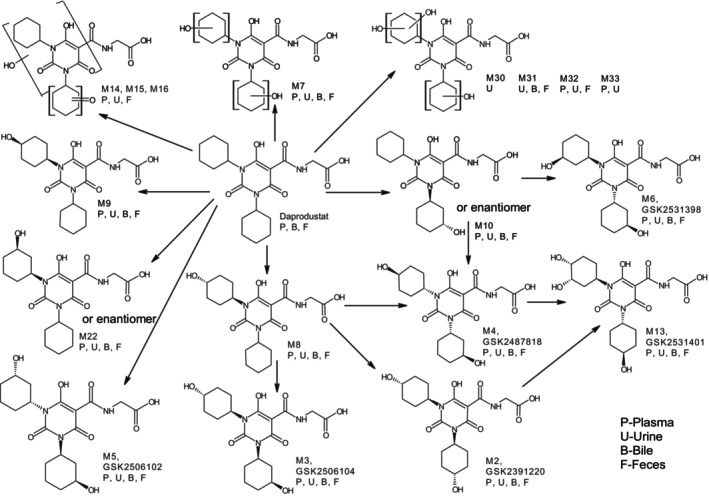
Proposed scheme of metabolites of [^14^C]‐daprodustat following administration to humans.

Notably, the presence of unchanged daprodustat in human feces and urine was low (approximately 0.5% of dose), whereas oxidative metabolites, essentially the same as those in plasma, accounted for the vast majority of radioactivity. In addition, the radio‐metabolic profile in human duodenal bile following a microtracer IV infusion of [^14^C]‐daprodustat (TP1) was very similar to that observed in feces following oral administration, suggesting that a substantial majority of the oral dose was absorbed, cleared exclusively by oxidative metabolism, and eliminated via hepatobiliary and urinary excretion. Most of the metabolites were formed by oxidation of the two cyclohexane rings. A proposed metabolic scheme is shown in Figure 5.

Five of the six predominant circulating human metabolites (M3, M4, M5, M6, and M13) contain chiral centers and could therefore exist in multiple stereoisomeric forms (M2 does not contain a chiral center). In a previous analysis of human urine samples (data not shown), metabolites M2, M3, M4, and M13 were determined to be present as single stereoisomers (i.e., GSK2391220 [M2, achiral], GSK2506104 [M3], GSK2487818 [M4], and GSK2531401 [M13]). Metabolites M5 and M6 were detected as pairs of stereoisomers consisting of GSK2506102 (79%) and GSK2531399 (21%) for M5, and GSK2531398 (89%) and GSK2531407 (11%) for M6. Further chiral analysis of metabolites M3 and M13 in plasma samples (data not shown) confirmed that the predominant circulating human stereoisomers of M3 and M13 were GSK2536104 and GSK2531401, respectively. The defined predominant stereoisomeric form for each metabolite (i.e., GSK2391220 for M2 [achiral], GSK2506104 for M3, GSK2487818 for M4, GSK2506102 for M5, GSK2531398 for M6, and GSK2531401 for M13) has been used in the safety evaluations and method development and validation of achiral bioanalytical assays.

In human hepatocytes, [^14^C]‐daprodustat was metabolized to mono‐ and di‐oxygenated products, formed by the hydroxylation of the cyclohexane rings (data not shown). CYP2C8 was the major enzyme responsible for oxidative metabolism and further confirmed by a gemfibrozil drug interaction study in human.^22^ All metabolites detected in human hepatocytes were also detected in rabbit and monkey hepatocytes, suggesting that no human‐specific metabolites were formed. Furthermore, the current radiolabel ADME study did not detect any new metabolites at significant levels, either in circulation or in renal and fecal excreta.

It is worth mentioning that quantitation of more than a dozen circulating DRMs can be challenging due to limitations of instrument sensitivity. In the current study, the quantitative measurement of plasma radioactive peaks on a conventional TopCount microplate counter was enabled through robust multiple HPLC injection/cumulative fraction collection. With a single injection, the LLQ would have been 6% of the total sample radioactivity for the 0–8 h plasma pool, and the majority of the quantified peaks reported in the current study would have been undetected or at least below the LLQ. Since the analytical sensitivity is in proportion to the number of injections, this multiple HPLC injection approach has lowered the LLQ to 1%, and greatly improved the accuracy of daprodustat metabolite profiling. Along with the deconvolution method for the determination of the individual level of M2, it ensured confirmation that all predominant metabolites were less than the threshold of 10% of total DRM in human plasma after a single dose to healthy subjects.

The inclusion of duodenal bile sampling in human ADME studies is still a relatively rare activity as, historically, bile collection was done using an invasive approach.^23^, ^24^ With the advent of a noninvasive approach, such as the bile string devices used in this investigation, along with the insights, it can provide into routes of metabolism and clearance that would otherwise be undiscovered, has meant that this is becoming either routine, as it is at GSK^25^, ^26^, ^27^, ^28^ or considered for inclusion more commonly.

In summary, following oral dosing of radiolabeled daprodustat, a large proportion of the dose was absorbed, primarily cleared by oxidative metabolism and eliminated via hepatobiliary and urinary excretion. Unchanged daprodustat was the principal circulating DRM with each of the six predominant metabolites individually representing <10% of DRM.

## AUTHOR CONTRIBUTIONS

Liangfu Chen and Guoying Tai contributed to the concept and design, performed data analysis, and helped to write the draft. Fangming Xia conducted experiments, contributed new reagents or analytic tools, and performed data analysis. Cathy Chen contributed to the concept and design, conducted experiments, contributed new reagents or analytic tools, performed data analysis, and helped to write the draft. Adrian Pereira conducted experiments and performed data analysis. Jill Pirhalla and Graeme Young contributed to the concept and design. Xiusheng Miao and Claire Beaumont performed data analysis. All authors provided critical revision for important intellectual content and agree to be accountable for this work. Artificial intelligence (AI)‐assisted technologies (large language models, chat bots, or image creators) were not used in the production of the submitted work.

## CONFLICT OF INTEREST STATEMENT

Liangfu Chen, Cathy Chen, Adrian Pereira, Xiusheng Miao, Graeme Young, and Guoying Tai are employees of and hold stock in GSK. Fangming Xia, Claire Beaumont and Jill Pirhalla are former employees of GSK.

## ETHICS STATEMENT

The clincial study was approved by an independent ethics committee and was conducted according to the recommendations of Good Clincial Practice and the Declaration of Helsinki. All subjects provided written informed consent to participate in the study.

## Supporting information


Data S1.

## Data Availability

Anonymized individual participant data and study documents can be requested for further research from https://www.gsk‐studyregister.com/en/.
