# Supplementary material for: Investigation of the human metabolism and disposition of the prolyl hydrolase inhibitor daprodustat using IV microtracer with Entero‐Test bile string
Source: Pharmacol Res Perspect. 2023 Oct 26;11(6):e1145. doi: 10.1002/prp2.1145 (PMC10603292; doi:10.1002/prp2.1145)
Supplement: Supplementary file 1 — Data S1. [file PRP2-11-e1145-s001.docx]

**Supplemental Materials**

**Supplemental item 1. Preparation of Cold Standard Mix Solution**

Primary Stock Solutions

Cold standards of daprodustat and six metabolites (M2, M3, M4, M5, M6, and M13) were weighed (approximately 5 mg each) directly into separately labelled screw cap glass scintillation vials. A purity corrected volume of anhydrous N,N-Dimethylformamide was added to each vial with vortex mixing to give a final concentration of 5.0 mg of free base/mL for primary stock solution. The primary stock solutions were stored at approximately 4˚C in the dark prior to use.

Secondary Stock Solutions

Each primary stock solution was diluted in a 2-mL screw cap polypropylene tube by mixing 10 µL with 990 µL of HPLC-grade water to give a final concentration of 50 µg/mL.

Standard Mix Solution

A mix solution of all seven standards was prepared in a single 15-mL screw cap Falcon tube by mixing 25 µL of each secondary stock solution with 9825 µL of HPLC-grade water to give each component a final concentration of 12.5 µg/mL.

**Supplemental Item 2. Radio-HPLC Conditions**

The 1200 HPLC system (plasma, urine, and fecal extracts) and 1100 HPLC system (bile string extracts) (Agilent Technologies, Palo Alto, CA) consisted of a binary pump, an autosampler, and a column compartment. The HPLC column was a LUNA C18 5 µm 4.6 x 250 mm; (Phenomenex, Torrance, CA.) Aliquots of samples were injected and eluted at 1 mL/min with 10 mM ammonium formate, pH 3.0 adjusted with formic acid (solvent A) and acetonitrile (solvent B). The following gradient was used: 0-4 minutes, isocratic at 13% B; 4-60 minutes, 13%-95% B; 60-65 minutes, isocratic at 95% B; 65-66 minutes, 95%-13% B; 66-76 minutes, isocratic at 13% B.

**Radio-HPLC Conditions for the Analysis of [^14^C]-Daprodustat and Its Metabolites in Plasma, Urine and Fecal Extracts (LC-MS^n^)**

| Mobile Phase Pump: | Agilent 1200 Model G1312B | | |
| --- | --- | --- | --- |
| Mobile Phase A: | 10 mM Ammonium Formate pH 3.0 | | |
| Mobile Phase B: | Acetonitrile | | |
| Flow Rate: | 1.0 mL/min | | |
| Gradient: | Time (min) | %A | %B |
|  | 0 | 87 | 13 |
|  | 4 | 87 | 13 |
|  | 60 | 5 | 95 |
|  | 65 | 5 | 95 |
|  | 66 | 87 | 13 |
|  | 76 | 87 | 13 |
| Autosampler: | Agilent 1200 Model G1367C | | |
| Column Compartment: | Agilent 1200 Model G1316B | | |
| Column: | Phenomenex LUNA C18(2) 5µm 250x4.6mm | | |
| UV Detector (Wavelength): | Agilent 1290 DAD Model 4212A (266 nm) | | |
| Fraction Collector | ARC LCJet Model A | | |
| Fractionation Rate | 11.9 sec/well | | |

**Radio-HPLC Conditions for the Analysis of [^14^C]-Daprodustat and Its Metabolites in Duodenal Bile Extract (LC+AMS)**

| Mobile Phase Pump: | Agilent 1100 series binary pump | | |
| --- | --- | --- | --- |
| Mobile Phase A: | 10 mM Ammonium Formate pH 3.0 | | |
| Mobile Phase B: | Acetonitrile | | |
| Flow Rate: | 1.0 mL/min | | |
| Gradient: | Time (min) | %A | %B |
|  | 0 | 87 | 13 |
|  | 4 | 87 | 13 |
|  | 60 | 5 | 95 |
|  | 65 | 5 | 95 |
|  | 66 | 87 | 13 |
|  | 76 | 87 | 13 |
| Autosampler: | Agilent 1100 series | | |
| Column Compartment: | Agilent 1100 series | | |
| Column: Column Temperature: | Phenomenex LUNA C18 5µm 250x4.6mm 21 °C | | |
| UV Detector (Wavelength): | Agilent 1100 series (266 nm) | | |
| Fraction Collector: | Agilent 1100 series | | |
| Fractionation Condition: | 12 seconds/fraction between 15 and 55 minutes (200 fractions/chromatogram) | | |

**Supplemental Item 3. HPLC-UV+AMS Analysis for Duodenal Bile Samples**

Separate HPLC fractions and aliquots of injectate were dried in a blank quartz tube and combined with a second tube containing an aliquot (20 µL) of carbon carrier; dried aqueous sodium benzoate (a solution in HPLC grade water to a concentration of 90 mg/mL; ca 1.05 mg sodium benzoate) and copper oxide (ca 50 mg), both sealed into evacuated tapered quartz tubes and heated at 900°C for 2 h. The CO_2_ thus formed was cryogenically transferred into evacuated tubes, containing zinc powder, titanium hydride and cobalt catalyst, and the tube was sealed. This reduction tube was heated to 500°C for 4 h, followed by 550°C for 6 h to complete the graphitization process. Carbon as graphite, deposited on the cobalt, was pressed into aluminum cathodes and analyzed by AMS. Control samples, including Australian National University (ANU) sugar (ca 6.5 mg, n=6) and dried aliquots (20 µL, n=6) of aqueous sodium benzoate were processed with the run. Instrument standards of pooled ANU graphite and synthetic graphite were used to normalize the data and check suitability of instrumental background, respectively. The level of carbon present in the sodium benzoate solution was calculated on the basis of the molecular weight of the solid material which was assumed to be 100% pure (i.e., 58 % carbon). The data from the AMS and the carbon content of the sodium benzoate solution were combined to provide radiocarbon levels for each sample.
